# Loss of Ift74 Leads to Slow Photoreceptor Degeneration and Ciliogenesis Defects in Zebrafish

**DOI:** 10.3390/ijms22179329

**Published:** 2021-08-28

**Authors:** Panpan Zhu, Jingjin Xu, Yadong Wang, Chengtian Zhao

**Affiliations:** 1 Institute of Evolution & Marine Biodiversity, Ocean University of China, Qingdao 266003, China; zhupanpan@ouc.edu.cn (P.Z.); xujingjin@stu.ouc.edu.cn (J.X.); yadong@uw.edu (Y.W.); 2 Laboratory for Marine Biology and Biotechnology, Qingdao National Laboratory for Marine Science and Technology, Qingdao 266003, China; 3 Sars-Fang Centre, Ministry of Education Key Laboratory of Marine Genetics and Breeding, College of Marine Life Sciences, Ocean University of China, Qingdao 266003, China

**Keywords:** zebrafish, *ift74*, cilia, photoreceptor degeneration, opsin transport

## Abstract

Cilia are microtubule-based structures projecting from the cell surface that perform diverse biological functions. Ciliary defects can cause a wide range of genetic disorders known collectively as ciliopathies. Intraflagellar transport (IFT) proteins are essential for the assembly and maintenance of cilia by transporting proteins along the axoneme. Here, we report a lack of Ift74, a core IFT-B protein, leading to ciliogenesis defects in multiple organs during early zebrafish development. Unlike rapid photoreceptor cell death in other *ift-b* mutants, the photoreceptors of *ift74* mutants exhibited a slow degeneration process. Further experiments demonstrated that the connecting cilia of *ift74* mutants were initially formed but failed to maintain, which resulted in slow opsin transport efficiency and eventually led to photoreceptor cell death. We also showed that the large amount of maternal *ift74* transcripts deposited in zebrafish eggs account for the main reason of slow photoreceptor degeneration in the mutants. Together, our data suggested Ift74 is critical for ciliogenesis and that Ift proteins play variable roles in different types of cilia during early zebrafish development. To our knowledge, this is the first study to show *ift-b* mutant that displays slow photoreceptor degeneration in zebrafish.

## 1. Introduction

Retinal degeneration is one of the major causes of human blindness due to progressive death and dysfunction of photoreceptors. The vertebrate photoreceptor cell is a specialized type of neuroepithelial cell with a distinctive morphology, consisting of an outer segment (OS), inner segment (IS), the nuclear region, and synapse. OS can be recognized as a highly modified sensory cilium that is rich in the photosensitive G protein-coupled receptors (GPCRs), opsins. The OS and IS are connected through a narrow structure called the connecting cilium, through which opsin and other OS protein components synthesized in the IS can be transported into the OS [1,2].

Cilia are highly conserved organelles extending from the surface of almost all vertebrate cells. Generally, cilia are often classified as either motile or primary (sensory) cilia.

Motile cilia in the respiratory tract help the transport of mucus toward the throat, and movement of ependymal cilia creates a flow of cerebrospinal fluid in the brain ventricles. The primary cilia function as cellular antenna in that they are enriched for multiple membrane receptors for sensing extracellular signals, such as growth factors, hormones, odor molecules, and developmental morphogens [3,4,5]. Defects in cilia formation and/or function can lead to a wide range of inherited ciliopathic diseases in humans, including retinal degeneration, kidney disease, brain abnormalities, *situs inversus*, and other defects [6].

The assembly, maintenance, and function of the cilia require bidirectional movement of protein complexes along the microtubule-based axonemes. Ciliogenesis requires intraflagellar transport (IFT) to mediate the delivery of components such as tubulin, dyneins, and membrane proteins to the tip of the cilia, where new cilia assembly occurs [7]. The IFT particle is formed by two complexes, complex A and complex B, consisting of at least 6 and 14 subunits, respectively. In the anterograde direction, the IFT complex binds to kinesin-2 heterotrimer through IFT-B to transport related substances to the tip of cilia, while transport from cilia tip to base (retrograde) is mediated by IFT complex A (IFT-A) and the cytoplasmic dynein motor [7,8]. In vertebrates, the antegrade Kinesin-2 family contains four members: KIF3A, KIF3B, KIF3C, and KIF17. While KIF17 forms homodimers for fast, highly processive transport, KIF3A and KIF3B are normally found in a heterotrimeric complex together with the kinesin-associated protein 3 (KAP3) [9]. In zebrafish and mouse, KIF3 heterotrimer motors play a major role during ciliogenesis as cilia are still present in mutants lacking KIF17 proteins [9,10,11].

Being a modified cilium, it is easy to speculate that mutations of either IFT components or Kinesin motors can affect the formation of outer segments, thus interrupting the function of photoreceptor cells and leading to visual loss. The conditional knockout of KIF3A in a mouse photoreceptor cell layer results in dysplasia of the connecting cilia, which in turn lead to apoptosis of rods and cones [12,13]. In zebrafish, both *kif3a* and *kif3b* mutants display abnormal photoreceptor development at early developmental stages. Interestingly, rod photoreceptors degenerate rapidly while cones undergo a slow degenerate process in these kinesin-2 mutants. An abnormal accumulation of opsins in the rods cell membrane due to insufficient transport is the major cause of fast rods degeneration [11]. IFT plays a critical role during OS assembly and mutations in genes encoding IFT proteins, such as IFT88 and IFT172, causing severe ablation or disruption of OS [14,15,16,17]. Of note, the photoreceptor degeneration process is less severe in the mutants of IFT-A components than those of IFT-B components, which is consistent with the concept that components of the IFT-B subunit are essential for ciliogenesis [18].

IFT74, together with its short protein variant, IFT72, forms a heterotetramer with IFT81, composing the backbone of the IFT-B core complex. The interaction between IFT74 and IFT81 proteins forms a module that is essential for the binding of tubulin, the backbone, and the most abundant ciliary proteins [19]. Recently, *IFT74* has been identified as causative for human ciliopathies, Bardet–Biedl syndrome (BBS), and Joubert syndrome (JBTS) [20,21,22]. The role of IFT74 during embryonic development remains to be elucidated. Here, we identified zebrafish *ift74* mutants and reported ciliogenesis defects in most organs. Unexpectedly, mutant photoreceptor cells displayed slow onset progressive degeneration as late as 5 days post fertilization, which is different from the rapid photoreceptor degeneration occurring in other IFT-B core complex mutants. We further examined the reasons for slow photoreceptor degeneration in *ift74* mutants and uncovered variable requirements of maternal proteins during early ciliogenesis.

## 2. Results

### 2.1. Zebrafish michelin Locus Encodes ift74, an IFT-B Component

**Figure 1:**
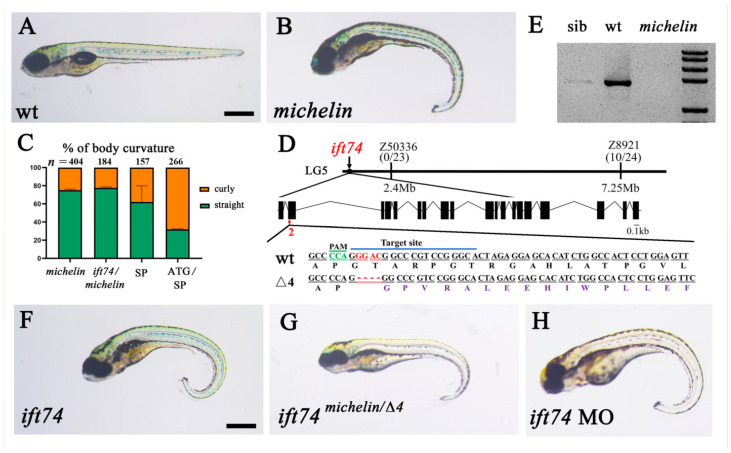
*michelin* encodes a zebrafish homolog of the *ift74* gene. (**A**,**B**) External phenotypes of wild-type (wt) and *michelin* mutants at 5 dpf. (**C**) Bar graph showing the percentages of embryos with body curvature defects in different groups as indicated. SP, splicing morpholino; ATG, translation-blocking morpholino. (**D**) Genetic map on LG 5 showing the number of recombinants near *michelin/ift74* locus. The exon/intron structure of the *ift74* transcript was shown in the middle. Bottom showing the sequence of wild-type and *ift74* mutants generated with CRISPR/Cas9 methods. The sgRNA targeting site was indicated with overline, the PAM site was labeled in green and the changing bases in the target region were shown in red. (**E**) PCR analysis showing the amplification of *ift74* ORF in 3 dpf wild-type, sibling, and *michelin* mutant larvae. (**F**–**H**) External phenotypes of 5 dpf *ift74* mutant and morphant larvae as indicated. Scale bars: 500 µm.

The *michelin* mutant is a spontaneous line occurring in our lab, and the mutant is characterized by the presence of a ventral body axis (Figure 1A,B). We initially named this line as *michelin* because the bended tail resembles a tire. Approximately 25% of the produced embryos from two intercrossed *michelin* heterozygotes showed body curvature phenotype (Figure 1C), suggesting that these mutants were caused by the mutation of a single allele. Next, we performed linkage analysis and mapped the mutation to a region in linkage group 5 (LG5) defined by microsatellite Z8921 (10 recombination in 24 meiotic events) and Z50336 (0 recombination in 23 meiotic events, Figure 1D). We found that *ift74*, encoding a component of the IFT-B core complex, is located in this region. To assess whether *michelin* harbors a mutation in the *ift74* gene, we performed PCR analysis to amplify the open reading frame (ORF) of the *ift74* gene from wild-type, *michelin* mutant, and their sibling embryos. The results showed that no band can be amplified from *michelin* homozygotes (Figure 1E). Further PCR results from genomic DNA showed that only the first exon was still present, while the remaining exons were absent in the mutant genome (Figure S1A). These results imply that there may have a chromosomal rearrangement occurring in the *ift74* locus in *michelin* mutants, which interrupts the transcription of the *ift74* gene.

To further prove that the mutation of *ift74* can result in *michelin* mutant phenotypes, we generated zebrafish *ift74* mutants with the CRISPR/Cas9 method. The sgRNA targeting the second exon of *ift74* was used to introduce a double-stranded break, which led to genetic mutations due to subsequently non-homologous end-joining (NHEJ) repair. We recovered a mutant line carrying 4 bp deletion in the target region, causing a frameshift mutation of the *ift74* gene during mRNA translation (Figure 1D). The expression of mutant messenger RNA was also decreased in the mutants due to nonsense mediated decay, which further confirmed the specificity of this mutant (Figure S1B,C). Similar to *michelin* mutants, mutation of the *ift74* gene also led to strong body curvature defects (Figure 1F). In addition, crossing between *michelin* and *ift74* heterozygotes also produced approximately 25% (42 in 184) of embryos with body curvature defects (Figure 1C,G). Finally, knocking down of *ift74* with two individual morpholinos to block translation (ATG-MO) and splicing (SP-MO) also caused embryos to develop a ventral curly body axis (Figure 1C,H). Taken together, these results provide convincing evidence that *michelin* encodes *ift74*. In the following study, we performed all analysis using *ift74* mutants.

### 2.2. Ciliary Defects in Multiple Organs of ift74 Mutants

**Figure 2:**
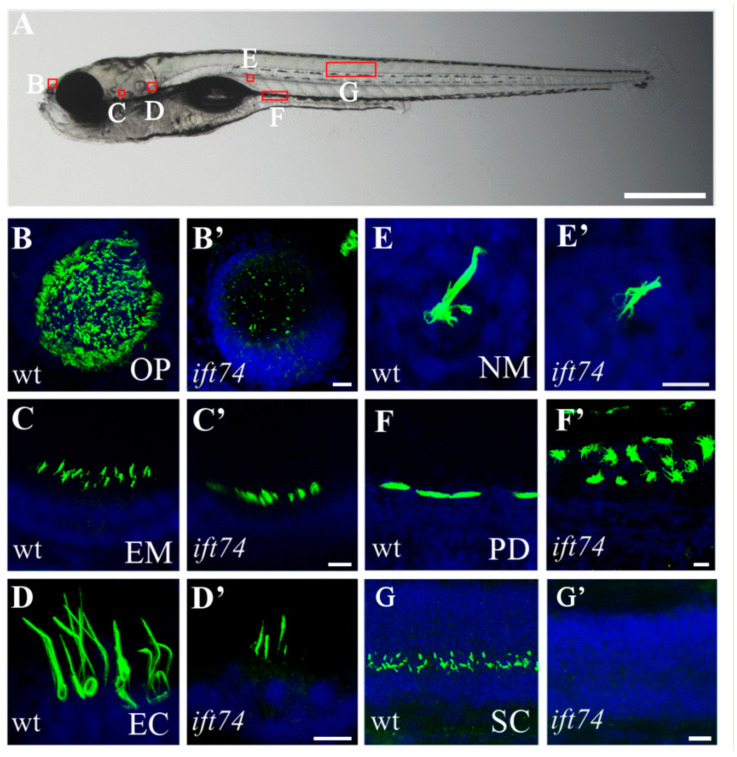
Cilia defects in *ift74* mutants. (**A**) Representative image showing the position of cilia in different organs as indicated in a 5 dpf zebrafish larva. (**B**–**G′**) Confocal images showing cilia in different organs of wild-type and *ift74* mutants as indicated. Cilia were visualized with anti-glycylated tubulin antibody (green), and nuclei were counterstained with DAPI (blue). OP, olfactory placode; EM, ear macula; EC, ear crista; NM, neuromast; PD pronephric duct; SC, spinal canal. Scale bars: (**A**), 500 µm; (**B′**–**G′**), 10 µm.

Considering that Ift74 is one of the core IFT-B complex required for ciliogenesis, we first analyzed cilia development in *ift74* mutants. Cilia are present in diverse organs of developing zebrafish, including olfactory placode, pronephric duct, ventral spinal cord, ear, photoreceptor, and neuromasts (Figure 2A) [23]. To directly visualize cilia, we performed whole-mount immunocytochemistry with anti-glycylated α-tubulin antibody on 3 and 5 days post fertilization (dpf) zebrafish larvae. Compared with those in sibling embryos, cilia displayed various defects in *ift74* mutants (Figure 2 and Figure S2). Cilia in nasal pit and spinal cord were nearly absent in the mutants, while cilia in hair cells of ear macula, ear crista, and neuromast are still present but shorter. In the pronephric duct, multicilia were shorter and disorganized in *ift74* mutants (Figure 2 and Figure S2). These results suggest that Ift74 is essential for the development of cilia in all examined organs.

### 2.3. Slow-Progressing Photoreceptor Cell Degeneration in ift74 Mutant

**Figure 3:**
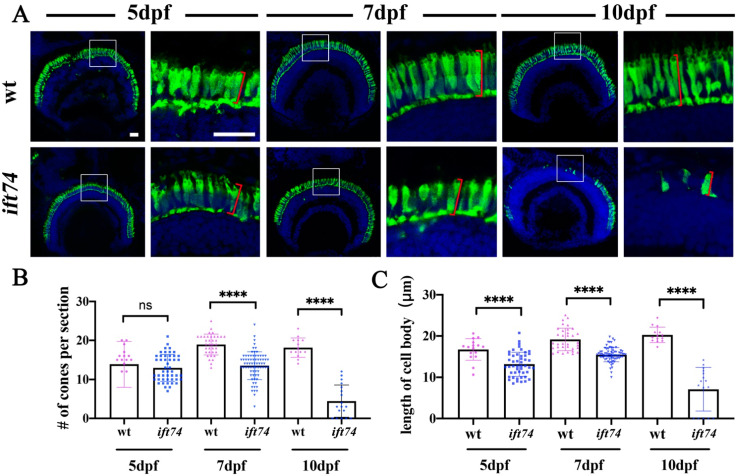
Slow photoreceptor degeneration in *ift74* mutants. (**A**) Confocal images showing the cell bodies of red and green double cones in wild-type and *ift74* mutant larvae at different stages as indicated. The double cones were stained with zpr-1 antibody (green), and nuclei were stained with DAPI (blue). Enlarged views of the boxed area are shown on the right. (**B**,**C**) Statistical results showing the number of cones per retina section and length of cone cell body in different groups as indicated. Measurements of the cell body length are shown in panel (**A**) (Red square brackets). Scale bars: 20 µm. **** *p* < 0.0001, ns, no significant.

Photoreceptor outer segments are highly modified sensory cilia for phototransduction. We examined photoreceptor development in *ift74* mutants. Immunostaining results with zpr-1 antibody, which selectively labels cell bodies of the red- and green-sensitive double cones [24], showed that the numbers of double cones per retinal cross-section were comparable between wild-type and mutant larvae at 5 dpf (Figure 3A,B). At 7 dpf and 10 dpf, the number of double cones decreased significantly (Figure 3A,B). The staining was almost completely disappeared in the center retinae of 10 dpf mutant larvae, suggesting that the majority of cone photoreceptors degenerated at this time (Figure 3A,B). Meanwhile, the length of the cell body was significantly shorter in the mutants starting at 5 dpf (Figure 3C). We further evaluated outer segments formation in the mutants by staining with wheat germ agglutinin (WGA) or zpr3 antibody, which labels all the outer segments or rod outer segments, respectively. Similarly, photoreceptor outer segments were initially formed at 5 dpf, while they degenerated during development in both *ift74* and *michelin* mutants (Figure S3). These results suggested that photoreceptors were initially formed and endured slow progressive degeneration in the absence of Ift74 protein.

Considering that cilia were defective in multiple organs as early as 3 dpf, the slow photoreceptor degeneration in the mutants was quite surprising, especially when multiple mutant lines of the IFT-B complex have been reported to exhibit severe photoreceptor degeneration at 5 dpf [14,16,25]. Therefore, we compared photoreceptor survival between *ift74*, *ift88,* and *kif3a* mutants. Ift88 is a member of the IFT-B complex and Kif3a is the major motor protein required for IFT in vertebrate cilia. In contrast to the slow progressive degeneration in *ift74* mutants, both *ift88* and *kif3a* mutants displayed rapid photoreceptor degeneration at 5 dpf zebrafish larvae (Figure S4).

### 2.4. Ift74 Is Required for the Maintenance of Connecting Cilium

**Figure 4:**
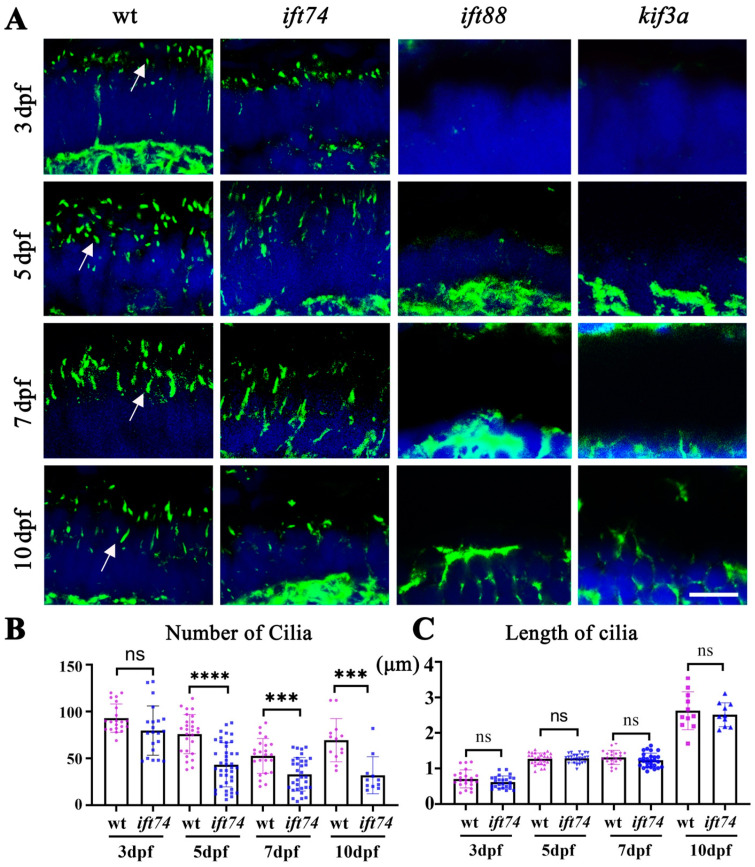
Photoreceptor connecting cilia in *ift74*, *ift88,* and *kfi3a* mutants. (**A**) Confocal images showing photoreceptor connecting cilia in the retinae of wild-type and mutant larvae at different stages as indicated. Cilia were stained with anti-acetylated alpha tubulin antibody (green). Arrows indicate the connecting cilia. (**B**,**C**) Dot plots showing the number and length of connecting cilia in different groups as indicated. Scale bar: 10 µm. *** *p* < 0.001, **** *p* < 0.0001, ns, no significant.

To understand the reasons for the slow progressive degeneration of photoreceptor cells in *ift74* mutants, we evaluated the formation and maintenance of photoreceptor connecting cilium. Immunostaining results using anti-acetylated α-tubulin antibody showed that the number and length of connecting cilia were grossly normal in the retinae of 3 dpf mutant larvae (Figure 4A–C). At 5 dpf, the number of connecting cilia began to decrease significantly and was gradually lost during development (Figure 4B). Notably, the remaining cilia in the mutant photoreceptors were still of similar length with those in wild-type larvae (Figure 4C). In contrast, connecting cilia were absent in both *ift88* and *kif3a* mutants (Figure 4A). These results further confirmed that photoreceptor connecting cilia were initially formed but failed to maintain in *ift74* mutants, which is different from severe ciliogenesis defects in *ift88* and *kif3a* mutants.

### 2.5. Defects of Opsin Transport Machinery in ift74 Mutant Retina

**Figure 5:**
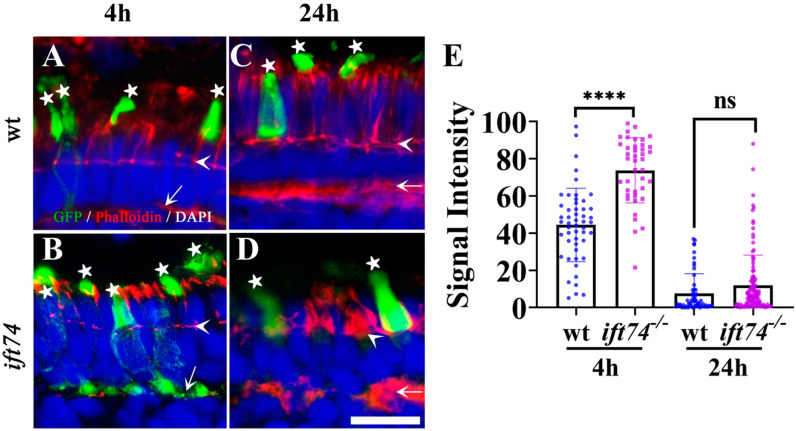
Opsin transport analysis in *ift74* mutant. (**A**–**D**) Representative confocal images of transverse cryosections through the central retinae of wild-type and *ift74* mutant larvae at 4 and 24 h after heat-shock induction. The subcellular distribution of the GFP-CT44 (green) was evaluated. Sections were counterstained with phalloidin (red) to visualize the outer limiting membrane and the outer plexiform layer. Cell nuclei were stained with DAPI in blue. (**E**) Relative GFP fluorescence intensity in the cell bodies measured between the outer limiting membrane and the outer plexiform layer. Asterisks indicate outer segments; arrowheads indicate the outer limiting membrane and arrows indicate the outer plexiform layer. Scale bar: 10 µm. **** *p* < 0.0001, ns, no significant.

Opsin mislocalization is one of the main causes of photoreceptor cell death. To determine the reasons for photoreceptor degeneration in the *ift74* mutant retinae, we further analyzed the transport efficiency of opsin. We performed a pulse-chase experiment by injecting zebrafish embryos with *hsp:GFP-CT44* construct, which encodes a GFP protein fused with the 44 C-terminal amino acids of xenopus opsin driven by a heat shock promoter. In photoreceptor cells, the GFP-CT44 chimeric protein is transported to the outer segment, which is similar to the localization of endogenous rhodopsin [26]. We injected this construct into one-cell stage zebrafish embryos collected from two intercrossed *ift74* heterozygotes. At 5 dpf, the injected embryos were heat induced for one hour at 37 °C. In control siblings, the majority of GFP signals were cleared from the inner segments and accumulated in the outer segments as early as 4 h after heat inducement (Figure 5A). In contrast, a large amount of GFP signals were still present in the inner segments of photoreceptor cells of the *ift74* mutants at 4 h after induction (Figure 5B). Interestingly, the chimeric protein became concentrated in the outer segments at 24 h after heat shock in both mutants and control siblings (Figure 5C–E).

Opsin mislocalization in the mutant photoreceptor cells may due to the overexpressed fusion proteins in some photoreceptors containing an excess amount of injected construct. To exclude such possibility, we further generated a stable line *Tg(hsp:GFP-CT44)* and crossed it with a transgenic line *Tg(sws1:HA-tdTomato-CT44)*. The *Tg(sws1:HA-tdTomato-CT44)* transgenic line contains a similar opsin localization signal fused to the C-terminal of tdTomato, which is driven by the promoter of the UV-sensitive opsin gene *sws1* [11,27]. We injected *ift74* morpholinos into the embryos carrying both transgenes. When measuring the GFP or tdTomato signals’ intensity, we found an ectopic localization of these chimeric proteins in the mutant inner segments at both 4 h and 24 h after heat shock (Figure S5). Taken together, these results suggest that opsin transport efficiency was compromised in both *ift74* mutants and morphants despite the presence of cilia at early stages.

### 2.6. Maternal Ift74 Proteins Contribute to the Early Development of Photoreceptors

**Figure 6:**
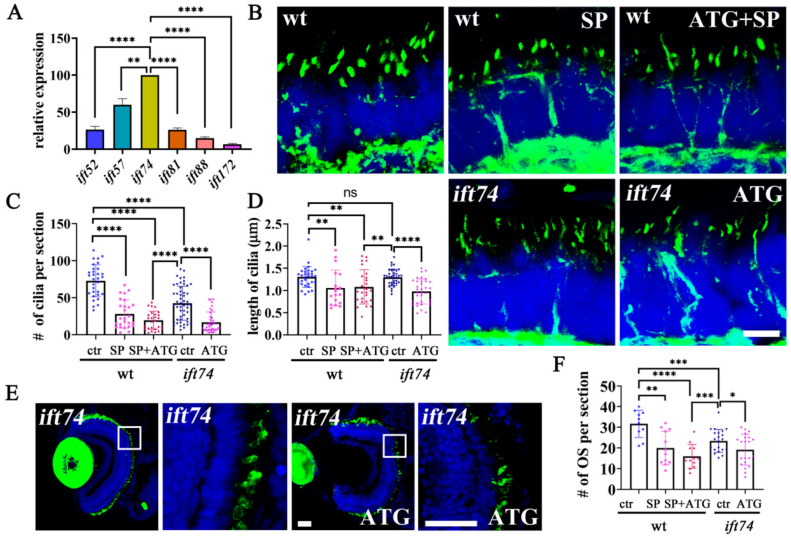
Maternal Ift74 contributes to the slow photoreceptor degeneration. (**A**) qRT-PCR results showing relative expression level of *ift-b* genes in one-cell stage embryos. The expression of the *ift74* gene was set as 100%. (**B**) Confocal images showing photoreceptor connecting cilia in the retinae of 5 dpf wild-type, *ift74* morphants, and mutant larvae. Cilia were stained with anti-acetylated alpha tubulin antibody (green). (**C**,**D**) Dot plots showing the number and length of connecting cilia in different groups as indicated. (**E**) Confocal images showing rod outer segments visualized with zpr-3 antibody in 5 dpf *ift74* mutants injected with control or ATG morpholino as indicated. Enlarged views of the boxed area are shown on the right. (**F**) Statistical results showing the number of outer segments per retina section in different groups as indicated. Scale bars: B, 5 µm; E, 30 µm. * *p* < 0.05, ** *p* < 0.01, *** *p* < 0.001, **** *p* < 0.0001, ns, no significant.

In *ift74* mutants, ciliogenesis defects occurred as early as 3 dpf in multiple tissues, including olfactory epithelium, spinal cord, and pronephric duct (Figure S2). Considering that Ift74 is an essential member of the IFT-B core complex, it is quite unexpected that the connecting cilium initially developed in the mutants, only leading to slow progressing degeneration. We think it is possible that maternal Ift74 proteins may contribute to the initial development of photoreceptors. To test this hypothesis, we evaluated the expression of maternal *ift-b* genes (encoding members of the IFT-B complex) by real-time quantitative PCR (RT-qPCR) analysis. By using total RNA extracted from single cell stage embryos, we showed that *ift52*, *ift57*, *ift74*, *ift81, ift88,* and *ift172* were all expressed maternally. The expression level of maternal deposited *ift74* gene was significantly higher than those of other *ift-b* genes (Figure 6A). To prevent the expression of maternal genes, we used *ift74* morpholinos (ATG-MO) to block the translation of both maternal and zygotic *ift74* genes by targeting the translational start region. In wild-type larvae, knockdown of the *ift74* gene with ATG- and SP-morpholinos inhibited the development of connecting cilium, which is much more severe than those of *ift74* mutants (Figure 6B–D). Then, we injected ATG-MO into *ift74* mutants to block the translation of maternal *ift74* transcripts, which exacerbated the severity of connecting cilium development (Figure 6B–D). Furthermore, photoreceptor degeneration was also enhanced in the absence of maternal transcripts in *ift74* mutants (Figure 6E,F). Finally, opsin proteins were still present in the inner segments at 24 h after heat inducement in *ift74* morphants, which is different to those of *ift74* mutants (Figure 5 and Figure S5). Together, these results suggested that maternal deposit *ift74* genes may be the main reason of the slow progressing photoreceptor degeneration in the mutants.

## 3. Discussion

Being a key member of the core IFT-B complex, the role of Ift74 during intraflagellar transport has been extensively investigated, while less is known about its roles during early embryonic development. In this study, we reported the role of Ift74 protein during early zebrafish development. Mutation of the *ift74* gene led to body curvature defects, which is a characteristic phenotype of ciliary mutants. The formation of a curly body axis was caused by abnormal urotensin signals originated from cilia-driven cerebrospinal fluid flow [23,28]. In line with this, we found ciliogenesis defects in multiple organs in the mutants as early as 3 dpf (Figure S2). Noticeably, the severity of ciliary defects was different among these organs. Nearly all the cilia were absent in the spinal canal and olfactory epithelium, while cilia were maintained but shorter in ear hair cells and pronephric ducts, suggesting different requirement of Ift74 proteins in these tissues during cilia formation.

Unexpectedly, *ift74* mutants displayed slow progressing photoreceptor degeneration. Both rod and cone photoreceptors were grossly normal in the mutants at 3 and 5 dpf, but they gradually degenerated with development. Some photoreceptor cells can survive as late as 10 dpf in the mutant retinae. These phenotypes were very different from the rapid photoreceptor degeneration in *ift88* and *kif3a* mutants (Figure S4). In other IFT-B mutants (*ift57*, *ift70* and *ift172*), rapid photoreceptor degeneration also occurred at early stages [14,15,16,17]. To the best of our knowledge, this is the first IFT-B mutant that displays slow photoreceptor degeneration in zebrafish. Actually, the photoreceptor defects were similar to those observed in *ift122* mutants, an IFT-A component, while no body curvature defects were reported in *ift122* mutants, implying the different mechanisms in ciliogenesis defects between IFT-A and IFT-B mutants [18].

The survival of photoreceptors relies on the presence of photoreceptor connecting cilium, through which opsin proteins can be transported to the outer segment. Consistent to the slow photoreceptor degeneration, connecting cilium was also formed at early stages, while it failed to maintain in the mutants. Opsin mislocalization was one of the major causes for photoreceptor cell death. We previously showed that the failure of connecting cilium development led to an ectopic localization of rhodopsin in the inner segments, where it activated intracellular calcium signals and finally resulted in cell death [11]. The presence of connecting cilium ensured the transport of opsin proteins in the mutant retinae and thus prevented the degeneration of photoreceptors at early stages. On the other hand, transient and stable transgenic analysis using GFP-tagged opsin proteins showed that opsin transport efficiency was still compromised in the mutants. In the retinae of wild-type larvae, opsin proteins are transported quickly as the majority of opsin proteins started to accumulate in the outer segments as early as 4 h after heat inducement. In contrast, opsin remained in the inner segment of mutant photoreceptors at 4 h after inducement. Interestingly, opsin transport was still functional, as the majority of opsin can be transported to the outer segments at 24 h after heat inducement in the mutant retinae. Together, these data suggested that opsin transport was defective with lower transport efficiency in the *ift74* mutants, which may represent the major reason accounting for slow photoreceptor degeneration.

In the *ift74* mutants, ciliogenesis defects occurred in multiple organs as early as in 3 dpf zebrafish larvae. It is surprising that photoreceptor connecting cilia were formed and maintained at 5 dpf. Even at 7 dpf, the remaining connecting cilia were still of similar length to those of wild-type larvae. It is clear that the formation and maintenance of connecting cilia depend on Ift74 proteins, considering opsin transport defects in the mutant. Interestingly, we found that the expression level of maternal *ift74* transcripts was significantly higher than those of other *ift-b* genes in one-cell stage zebrafish embryos. Maternal message RNAs play essential roles during maternal to zygotic transition to ensure the normal development of zebrafish embryos. These maternal *ift* mRNAs ensure the proper development of cilia during early stages. For instance, cilia were present in zygotic *ift88* (*oval*) mutants at 24 h post fertilization, while all cilia were absent in the *MZift88* mutants lacking both maternal and zygotic Ift88 proteins [29]. The presence of photoreceptor connecting cilia may due to the excess deposit of *ift74* maternal transcripts compared with those of other *ift-b* genes. Further knocking down of maternal *ift74* genes with *ift74* ATG-MO exacerbated photoreceptor degeneration in the mutants, which confirmed the importance of maternal transcripts. Interestingly, only the connecting cilia can be maintained for a while in the mutants, while all other types of cilia were still defective at earlier stages. It is possible that the photoreceptor connecting cilia were shorter and stable to ensure the fast transport of opsin proteins, where only a small amount of maternal proteins were sufficient to perform such roles. While in other tissues, the maintenance of entire cilia requires many more Ift74 proteins. The lower opsin transport efficiency in the mutants further confirmed the minimum requirement of Ift74 proteins for photoreceptor survival. Moreover, it is possible that the Ift74 degradation rate was slower in the photoreceptor cells, leading to the presence of a large amount of Ift74 proteins than other tissues.

In humans, IFT74 deficiency has been implicated in several ciliopathies, including BBS and JBTS [20,21,22]. Recently, male sterility due to impaired flagellogenesis was also reported [30]. In contrast to the gross ciliary defects in zebrafish *ift74* mutants, the clinical manifestations of human patients with *IFT74* mutations were quite variable. BBS patients are characterized by retinal dystrophy, obesity, polydactyly, and renal dysfunction, while JBTS patients have a distinctive cerebellar and brain stem malformation called the molar tooth sign (MTS) [31,32]. Interestingly, both JBTS and BBS *IFT74* patients displayed retinal defects, suggesting an essential function of this protein during retinal development. The pleiotropicity of these ciliopathies may be caused by the variety of *IFT74* mutations. For instance, we found that the BBS variant of IFT74 (Δ561–600-IFT74) lost its binding ability with IFT27, which is a Small GTPase-like protein required for BBSome function [33,34], while the JBTS variant (p. Q179E) maintains its binding activity with IFT27 [20]. It is apparent that the IFT74 variants reported in humans are hypomorphic, while our zebrafish mutants are likely to be a null allele, causing ciliogenesis defects in almost every organ. In the future, it is worth generating zebrafish mutants harboring different *ift74* variants or transgenic lines expressing these human variants in *ift74* homozygous mutants, which may provide further answers for the subtle difference of these IFT74 variants in different organs.

In summary, we showed that Ift74 was necessary for ciliogenesis in zebrafish. Malfunction of Ift74 resulted in ciliogenesis defects in the majority of organs, while photoreceptor connecting cilia were initially formed and degenerated slowly in the mutants. Compared with other genes encoding IFT-B complex, zebrafish eggs deposit more maternal *ift74* transcripts, representing the main causes of slow photoreceptor degeneration of the mutants.

## 4. Materials and Methods

### 4.1. Zebrafish Strain

All experiments involving zebrafish strains were maintained at 28 °C on a 14 h light/10 h dark cycle. Embryos were raised at 28.5 °C in E3 medium (5 mM NaCl, 0.17 mM KCl, 0.39 mM CaCl_2_, 0.67 mM MgSO_4_, 0.1% methylene blue (Sinopharm, Qingdao, China)) following standard protocols. CRISPR/Cas9 technology was used to generate zebrafish *ift74* mutants with the following target site for single guide (sg) RNA (5′-GTGCCCGGACGGGCCGTCCC-3′). The sgRNA was designed using ZiFit targeter (http://zifit.partners.org/ZiFiT/CSquare9Nuclease.aspx) (accessed on 5 December 2017). The Cas9 mRNA and sgRNA synthesis were similar to previously described [28,35]. Guide sgRNAs and Cas9 mRNA were co-injected into zebrafish embryos at one-cell stage. 

### 4.2. Genetic Mapping and Cloning

A map cross was set up between heterozygous carriers of *michelin* allele (Tübingen genetic background) and wild-type IND strain homozygotes. The *michelin* locus was mapped in a F2 intercross using bulked segregant analysis. DNA samples were genotyped by PCR with SSLP markers evenly spaced across zebrafish genome. To determine the *michelin* locus, we used a panel of 24 F2 diploid embryos obtained via incrossing of F1 adult animals. To determine the segregation patter of genomic polymorphisms, DNA samples were genotyped by PCR with SSLP markers evenly spaced across the F2 embryos’ genome. PCR products were electrophoresed on 4% agarose gels.

### 4.3. Whole-Mount In Situ Hybridization and Immunohistochemistry

For in situ hybridization, *ift74* gene was cloned from a wild-type cDNA library into pGEM-T vector, linearized with a restriction enzyme, and then transcribed using SP6 polymerase and digoxigenin-labeled UTP (Roche, Mannheim, Germany). Probe preparation and hybridization were carried out using standard protocols. For immunohistochemistry, zebrafish larvae were fixed in 4% paraformaldehyde (Sigma-Aldrich, Saint Louis, MO, USA) in phosphate-buffered saline (PBS, Sangon Biotech, Shanghai, China) overnight at 4 °C. Fixed larvae were cryoprotected using 30% sucrose in PBS, embedded in OCT (Leica, Nussloch, Germany), frozen, and sectioned at 12 µm thickness. Sections through the central retinae were placed on slides, dried for 2 h at 37 °C, rehydrated in PBST for 5 min, incubated in blocking solution (5% normal goat serum (G-clone, Beijing, China) and 0.5%Txiton X-100 (Sigma-Aldrich, Saint Louis, MO, USA) in PBST) for 30 min at room temperature, and incubated with primary antibodies in blocking solution overnight at 4 °C. Slides were washed with PBS three times for 10 min each and incubated with second antibodies for 2 h at room temperature. Antigen retrieval was performed on connecting cilia staining by incubating with 10 mM sodium citrate for 30 min at 65 °C, which was followed by regular immunostaining protocol. The following antibodies were used: anti-acetylated α-tubulin (1:500, Cell Signaling Technology, Danvers, MA, USA, #5335S), anti-glycylated tubulin (1:500, Millipore, Burlington, MA, USA), zpr-3 (1:500, Zebrafish International Resource Center, Eugene, OR, USA), zpr-1 (1:500, Zebrafish International Resource Center, Eugene, OR, USA). After staining, the confocal images were collected on a Leica TCS Sp8 confocal microscope.

### 4.4. Quantitative PCR

The primers used for qPCR are summarized in Supplemental Table S1. The PCR reaction was set up using Eva-Green Master Mix (ABM, Richmond, BC, Canada) and performed on a Step One real-time PCR system (Thermo Scientific, Waltham, MA, USA). The amplification condition parameters were 95 °C for 15 s, followed by 40 cycles of 95 °C for 5 s, 60 °C for 15 s, and 72 °C for 35 s, and each sample had a triplicate reaction. Relative gene express levels were quantified using the comparative Ct method (2^−∆∆Ct^ method) based on Ct values for target gene and zebrafish β-actin.

### 4.5. Morpholino Knockdown

To knockdown the expression of the *ift74* gene, we used a morpholino targeting to the 5′ untranslated region (ATG-MO) and an anti-splice site morpholino (SP-MO). These morpholinos were the same as described previously [20] (Table S1).

### 4.6. Opsin Transport Assay

The *hsp:GFP-CT44* chimeric construct was injected into *ift74* mutants or sibling embryos at the one-cell stage. For stable line analysis, we microinjected ATG and SP MOs into one-cell stage embryos collected from crossing between the *Tg(hsp:GFP-CT44)* and *Tg(sws1:HA-tdTomato-CT44)* transgenic line. The injected larvae were heat-shocked at 5 dpf for 1 h at 37 °C; then, they were fixed at 4 and 24 h after heat shock and cryosectioned with standard protocol. Sections were counter-stained with Alexa Fluor™ 546 Phalloidin(Invitrogen, Waltham, MA, USA) and imaged using a confocal microscope (SP8, Lecia, Wetzlar, Germany). Fluorescence signals were measured using ImageJ software(version1.53c, National Institutes of Health, Bethesda, MD, USA), and signals in the cell bodies were measured between the outer limiting membrane and the outer plexiform layer. The relative signal intensity of the cell body in each photoreceptor cell was calculated as percentages of fluorescence signal intensity between the cell body and entire cell.

### 4.7. Statistical Analysis

All experiments were repeated at least three times. Scatterplot data are presented as mean ± S.D. as indicated in the figure legends. Statistical significance was evaluated using Student’s *t*-test, and *p* < 0.05 was taken to be statistically significant. All statistical analysis was performed using GraphPad Prism 8 software(version 8.0.2, San Diego, CA, USA).

## Data Availability

The data presented in this study are available in article and Supplementary Material.

## Supplementary Materials

The following are available online at https://www.mdpi.com/article/10.3390/ijms22179329/s1. Figure S1: PCR analysis of *ift74* locus in *michelin* mutants, Figure S2: Cilia defects in 3dpf *ift74* mutants, Figure S3: Phenotypes of photoreceptor outer segments in *ift74* mutants, Figure S4: Rapid photoreceptor degeneration in *ift88* and *kif3a* mutants, Figure S5: Opsin transport defects in *ift74* morphants, Table S1: List of primers used in this study.

## References

[1] Barnes C.L., Malhotra H., Calvert P.D. (2021). Compartmentalization of Photoreceptor Sensory Cilia. Front. Cell Dev. Biol..

[2] Goldberg A.F., Moritz O.L., Williams D.S. (2016). Molecular basis for photoreceptor outer segment architecture. Prog. Retin. Eye Res..

[3] Song Z., Zhang X.L., Jia S., Yelick P.C., Zhao C.T. (2016). Zebrafish as a Model for Human Ciliopathies. J. Genet. Genomics.

[4] Garcia G., Raleigh D.R., Reiter J.F. (2018). How the Ciliary Membrane Is Organized Inside-Out to Communicate Outside-In. Curr. Biol..

[5] Hilgendorf K.I., Johnson C.T., Jackson P.K. (2016). The primary cilium as a cellular receiver: Organizing ciliary GPCR signaling. Curr. Opin. Cell Biol..

[6] Hildebrandt F., Benzing T., Katsanis N. (2011). Ciliopathies. N. Engl. J. Med..

[7] Wang W., Jack B.M., Wang H.H., Kavanaugh M.A., Maser R.L., Tran P.V. (2021). Intraflagellar Transport Proteins as Regulators of Primary Cilia Length. Front. Cell Dev. Biol..

[8] Lechtreck K.F. (2015). IFT-Cargo Interactions and Protein Transport in Cilia. Trends Biochem. Sci..

[9] Zhao C., Omori Y., Brodowska K., Kovach P., Malicki J. (2012). Kinesin-2 family in vertebrate ciliogenesis. Proc. Natl. Acad. Sci. USA.

[10] Jiang L., Tam B.M., Ying G., Wu S., Hauswirth W.W., Frederick J.M., Moritz O.L., Baehr W. (2015). Kinesin family 17 (osmotic avoidance abnormal-3) is dispensable for photoreceptor morphology and function. FASEB J..

[11] Feng D., Chen Z., Yang K., Miao S., Xu B., Kang Y., Xie H., Zhao C. (2017). The cytoplasmic tail of rhodopsin triggers rapid rod degeneration in kinesin-2 mutants. J. Biol. Chem..

[12] Lopes V.S., Jimeno D., Khanobdee K., Song X., Chen B., Nusinowitz S., Williams D.S. (2010). Dysfunction of heterotrimeric kinesin-2 in rod photoreceptor cells and the role of opsin mislocalization in rapid cell death. Mol. Biol. Cell.

[13] Marszalek J.R., Liu X., Roberts E.A., Chui D., Marth J.D., Williams D.S., Goldstein L.S. (2000). Genetic evidence for selective transport of opsin and arrestin by kinesin-II in mammalian photoreceptors. Cell.

[14] Sukumaran S., Perkins B.D. (2009). Early defects in photoreceptor outer segment morphogenesis in zebrafish ift57, ift88 and ift172 Intraflagellar Transport mutants. Vision Res..

[15] Krock B.L., Perkins B.D. (2008). The intraflagellar transport protein IFT57 is required for cilia maintenance and regulates IFT-particle-kinesin-II dissociation in vertebrate photoreceptors. J. Cell Sci..

[16] Tsujikawa M., Malicki J. (2004). Intraflagellar transport genes are essential for differentiation and survival of vertebrate sensory neurons. Neuron.

[17] Pazour G.J., Baker S.A., Deane J.A., Cole D.G., Dickert B.L., Rosenbaum J.L., Witman G.B., Besharse J.C. (2002). The intraflagellar transport protein, IFT88, is essential for vertebrate photoreceptor assembly and maintenance. J. Cell Biol..

[18] Boubakri M., Chaya T., Hirata H., Kajimura N., Kuwahara R., Ueno A., Malicki J., Furukawa T., Omori Y. (2016). Loss of ift122, a Retrograde Intraflagellar Transport (IFT) Complex Component, Leads to Slow, Progressive Photoreceptor Degeneration Due to Inefficient Opsin Transport. J. Biol. Chem..

[19] Bhogaraju S., Cajanek L., Fort C., Blisnick T., Weber K., Taschner M., Mizuno N., Lamla S., Bastin P., Nigg E.A. (2013). Molecular basis of tubulin transport within the cilium by IFT74 and IFT81. Science.

[20] Luo M., Lin Z., Zhu T., Jin M., Meng D., He R., Cao Z., Shen Y., Lu C., Cai R. (2021). Disrupted intraflagellar transport due to IFT74 variants causes Joubert syndrome. Genet. Med..

[21] Kleinendorst L., Alsters S.I.M., Abawi O., Waisfisz Q., Boon E.M.J., van den Akker E.L.T., van Haelst M.M. (2020). Second case of Bardet-Biedl syndrome caused by biallelic variants in IFT74. Eur. J. Hum. Genet..

[22] Lindstrand A., Frangakis S., Carvalho C.M., Richardson E.B., McFadden K.A., Willer J.R., Pehlivan D., Liu P., Pediaditakis I.L., Sabo A. (2016). Copy-Number Variation Contributes to the Mutational Load of Bardet-Biedl Syndrome. Am. J. Hum. Genet..

[23] Zhao L., Gao F., Gao S., Liang Y., Long H., Lv Z., Su Y., Ye N., Zhang L., Zhao C. (2021). Biodiversity-based development and evolution: The emerging research systems in model and non-model organisms. Sci. China Life Sci..

[24] Larison K.D., Bremiller R. (1990). Early onset of phenotype and cell patterning in the embryonic zebrafish retina. Development.

[25] Omori Y., Zhao C., Saras A., Mukhopadhyay S., Kim W., Furukawa T., Sengupta P., Veraksa A., Malicki J. (2008). Elipsa is an early determinant of ciliogenesis that links the IFT particle to membrane-associated small GTPase Rab8. Nat. Cell Biol..

[26] Zhao C., Malicki J. (2011). Nephrocystins and MKS proteins interact with IFT particle and facilitate transport of selected ciliary cargos. EMBO J..

[27] Takechi M., Hamaoka T., Kawamura S. (2003). Fluorescence visualization of ultraviolet-sensitive cone photoreceptor development in living zebrafish. FEBS Lett..

[28] Zhang X., Jia S., Chen Z., Chong Y.L., Xie H., Feng D., Wu X., Song D.Z., Roy S., Zhao C. (2018). Cilia-driven cerebrospinal fluid flow directs expression of urotensin neuropeptides to straighten the vertebrate body axis. Nat. Genet..

[29] Huang P., Schier A.F. (2009). Dampened Hedgehog signaling but normal Wnt signaling in zebrafish without cilia. Development.

[30] Lores P., Kherraf Z.E., Amiri-Yekta A., Whitfield M., Daneshipour A., Stouvenel L., Cazin C., Cavarocchi E., Coutton C., Llabador M.A. (2021). A missense mutation in IFT74, encoding for an essential component for intraflagellar transport of Tubulin, causes asthenozoospermia and male infertility without clinical signs of Bardet-Biedl syndrome. Hum. Genet..

[31] Bachmann-Gagescu R., Dempsey J.C., Phelps I.G., O'Roak B.J., Knutzen D.M., Rue T.C., Ishak G.E., Isabella C.R., Gorden N., Adkins J. (2015). Joubert syndrome: A model for untangling recessive disorders with extreme genetic heterogeneity. J. Med. Genet..

[32] Forsythe E., Kenny J., Bacchelli C., Beales P.L. (2018). Managing Bardet-Biedl Syndrome-Now and in the Future. Front. Pediatr..

[33] Eguether T., San Agustin J.T., Keady B.T., Jonassen J.A., Liang Y., Francis R., Tobita K., Johnson C.A., Abdelhamed Z.A., Lo C.W. (2014). IFT27 links the BBSome to IFT for maintenance of the ciliary signaling compartment. Dev. Cell.

[34] Liew G.M., Ye F., Nager A.R., Murphy J.P., Lee J.S., Aguiar M., Breslow D.K., Gygi S.P., Nachury M.V. (2014). The intraflagellar transport protein IFT27 promotes BBSome exit from cilia through the GTPase ARL6/BBS3. Dev. Cell.

[35] Chang N., Sun C., Gao L., Zhu D., Xu X., Zhu X., Xiong J.W., Xi J.J. (2013). Genome editing with RNA-guided Cas9 nuclease in zebrafish embryos. Cell Res..

