# Supplementary material for: Loss of Ift74 Leads to Slow Photoreceptor Degeneration and Ciliogenesis Defects in Zebrafish
Source: Int J Mol Sci. 2021 Aug 28;22(17):9329. doi: 10.3390/ijms22179329 (PMC8431285; doi:10.3390/ijms22179329)
Supplement: Supplementary file 1 [file ijms-22-09329-s001.zip › ijms-1332757-supplementary.pdf]

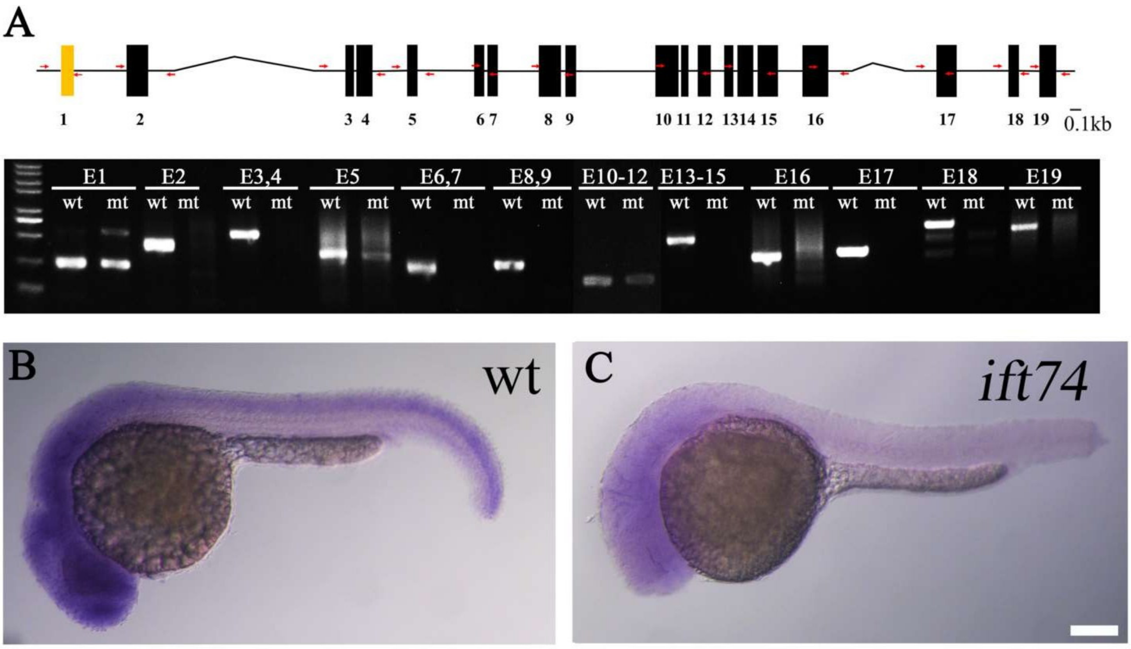

Fig S1 PCR analysis of *ift74* locus in *michelin* mutants.

(A) Top, genomic structure of the *ift74* gene. Bottom, PCR results showing amplification of different exons from wild-type and *michelin* genomic DNA. The first exon present in the mutant genome was shown in yellow. The positions of primers used for exon amplification were indicated with arrows on the top. The weak amplified bands with primer pairs E5, E10-12 and E16 in the mutants are non-specific amplification. (B,C) Whole mount in situ hybridization results showing the expression of *ift74* in wild-type and *ift74* mutant larvae as indicated. Scale bar: 200 µm.

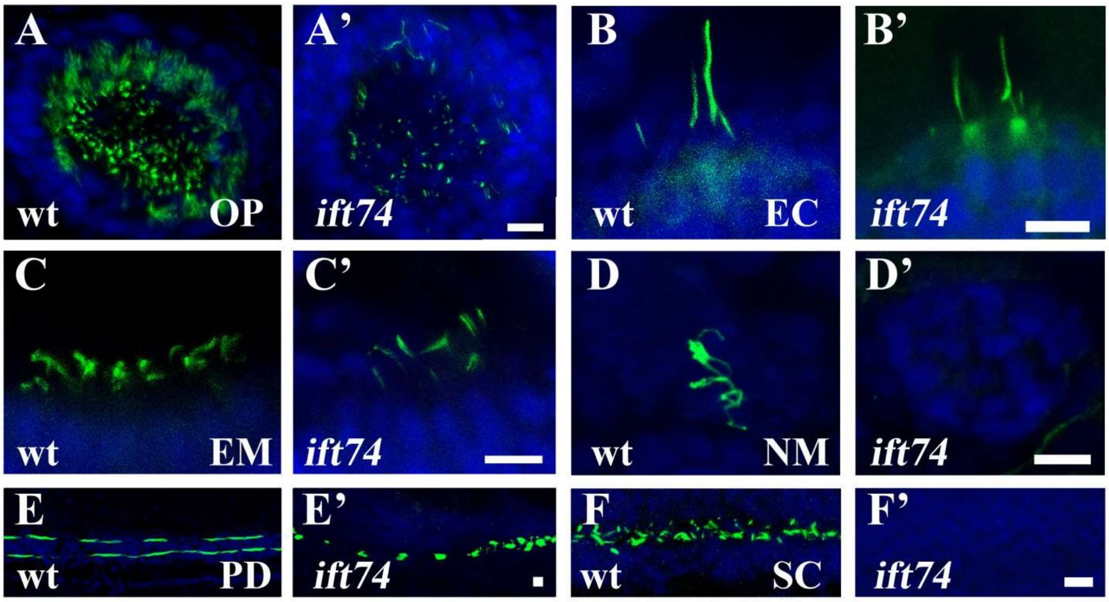

Fig S2 Cilia defects in 3dpf *ift74* mutants.

(A-F') Confocal images showing cilia in different organs of 3 dpf wild-type and *ift74* mutants as indicated. Cilia were visualized with anti-glycylated tubulin antibody (green) and nuclei were counterstained with DAPI (blue). OP, olfactory placode; EC, ear crista; EM, ear macula; NM, neuromast, PD pronephric duct; SC, spinal canal. Scale bars: 10 µm.

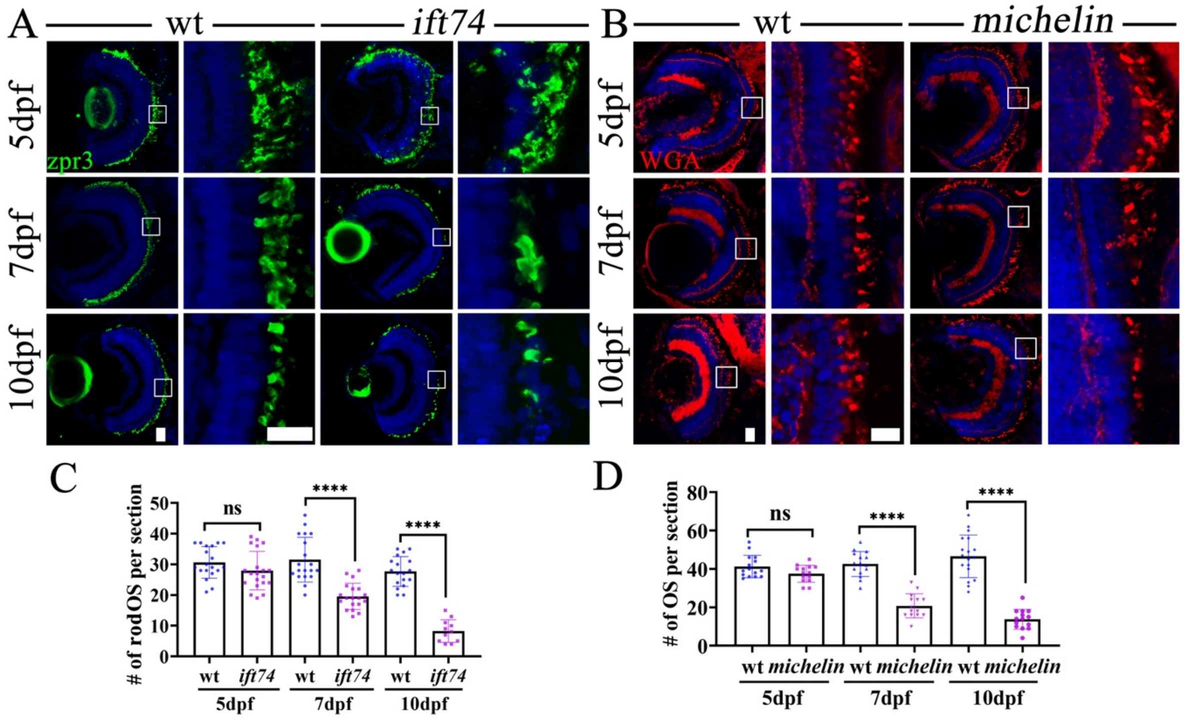

Fig S3 Phenotypes of photoreceptor outer segments in *ift74* mutants.

(A) Confocal images showing rod outer segments in the retinae of wild-type and mutant larvae at different stages as indicated. Outer segments were stained with zpr-3 antibody (green). (B) Confocal images showing the outer segments in the retinae of wild-type and mutant larvae at different stages as indicated. Outer segments were stained with WGA (red). Nuclei were counterstained with DAPI in blue. (C-D) Dot plots showing the number of rod outer segments and all the outer segments per retina section in different groups as indicated. Scale bars: 10 µm. **** P<0.0001, ns, no significant.

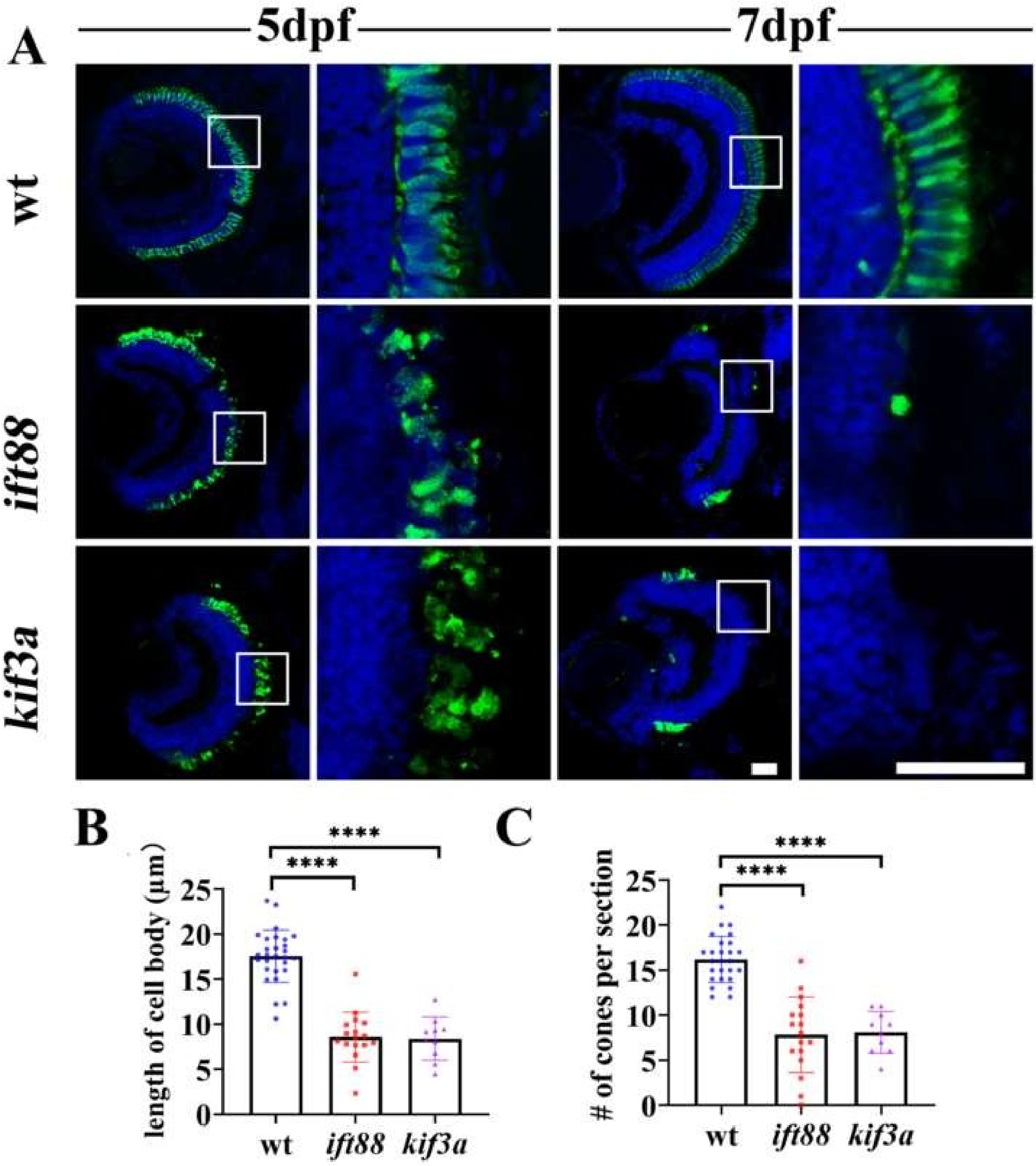

Fig S4 Rapid photoreceptor degeneration in *ift88* and *kif3a* mutants.

(A) Confocal images showing the cell bodies of red and green double cones in wild-type, *ift88* and *kif3a* mutant larvae at different stages as indicated. The double cones were stained with zpr-1 antibody (green) and nuclei were stained with DAPI (blue). Enlarged views of boxed area were shown on the right. (B-C) Statistical results showing the length and number of double cones in the retinae of 5dpf wild type and mutant larvae as indicated. Scale bars:10μm. *** P<0.001, **** P<0.0001.

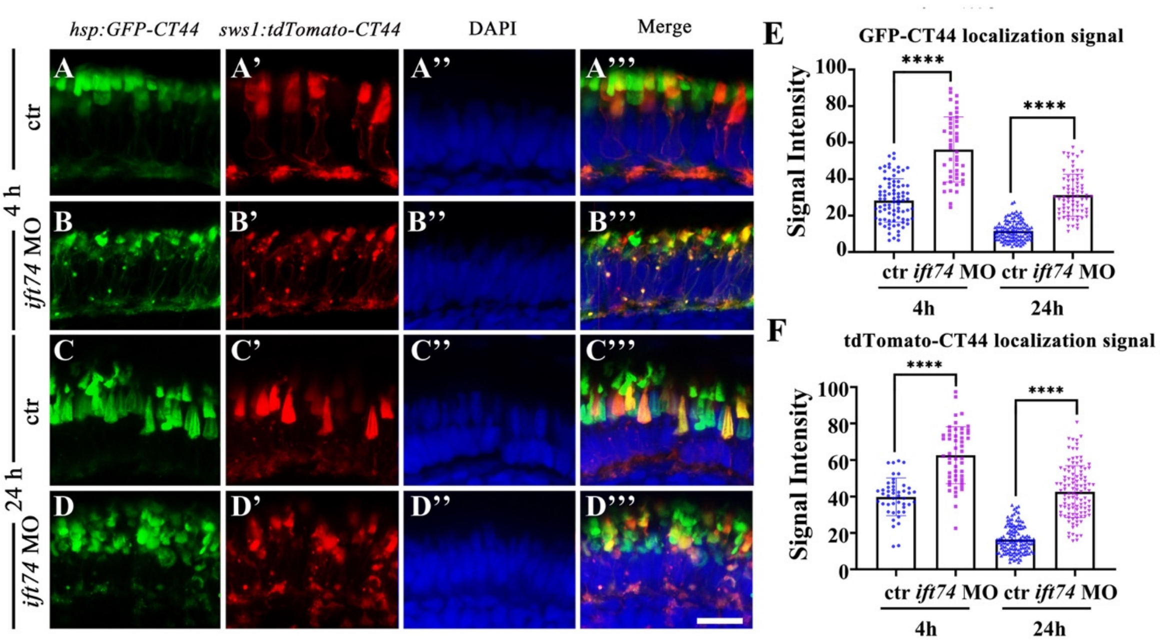

Fig S5 Opsin transport defects in *ift74* morphants.

Representative confocal images showing transverse sections through the central retinae of wild-type and morphant embryos at 4h (A-B''') and 24 h (C-D''') after heat-shock. The green channel shows the localization of GFP-CT44 driven by a heat shock promoter. The localization of tdTomato-CT44 driven by the *sws1* promoter was shown in red. The yellow signals indicate overlapping red and green signals. Sections were counterstained with DAPI (blue) to visualize cell nuclei. (E-F) Relative GFP fluorescence and tdTomato fluorescence intensity in the cell bodies measured between the outer limiting membrane and the outer plexiform layer. Scale bar: 10 μm. **** P<0.0001, ns, no significant.

Table S1 List of primers used in this study

**Primers for ORF amplification**	
primers	sequences
sense primer	P5: 5’-ATGTCGGCTCAGCGGCCGGCG-3’
antisense primer	P3: 5’-GCTCAGGGCCTCCAGCAGGAG-3’

**Primers for exon amplification**		
Exons	Sense primer	Antisense primer
Exon 1	ATAGACCTCACACCCGACATG	CACACACACACTCACCGCTGT
Exon 2	CTGACTGTGTGTAGTGTTCTGTC	TGACTTCGCCCACCACTACTC
Exon 3,4	ATCCACACAGCTCCTCATGTTTC	TGCAGCGGTGTGTGTTACTGATC
Exon 5	GTGCTGTTCTGAGGTCTAATTAC	CTCATATGCTGATTTGATCC
Exon 6,7	TTCTGCAGTTGGTGGACAAGC	TGTCGTCTCTGTGTGAAGATC
Exon 8,9	GCGGCTCAGGACATCATACAG	TCATCAGTGTGTCCAGCTCCT
Exon 10,11,12	GATCTCCTGCACTCTCAGGCT	CTTGATGTTCCTCCATGTCTGTG
Exon 13,14,15	GGGAACGAACTCAGAAGTACAAG	GTGCTCTGGGATTTCTGCATCTC
Exon 16	AGCTGGAGGGTAAGGTGTGTGTG	GAGCTGAAGGCCGCATGGATTGA
Exon 17	GTGTCCCCATGTAGTCTCCCATC	ATGCATGTAACCTGACCTCCGCG
Exon 18	TCAGAGGGCAGGATCCACACAAC	CCGTCCCAAATTAAACCCGTTAC
Exon 19	GCGTGGACATTTAAAGCAGTTAG	CTACACTCTGAGCTAACACACAC

**Sequences of *ift74* morpholinos**	
morpholino	sequence
ATG	5’-CGACATCACTGACAGAAAGTCAGCA-3’
SP	5’-GTCCTGCAAGAGTCAAGCACAGAGT-3’

**Primers for qPCR analysis**		
genes	Sense primers	Antisense primers
ift74	AGATTAAAGTGGCGGATCGTC	GATGTCATTCACCACTTCCTC
ift52	CTTCCACCACTCAAAGAACGC	GGAAGAGTGTTTGTGTGCAGC
ift81	GAGCAGACAGCCAAGAGAATG	CTCATACTGGTGGTAGGTCTC
ift88	GATTGGTACAGCTTATGGGTC	CTCTCTGTCTCACCAAAGCTC
ift57	CGGTGAGCAGTTCTATATGTT	CACACACTCATCCTCCACCTC
ift172	CTATGTGGTCAAGTCTATGGC	CAATGAAATACCGAACCACCG
β-actin	CCGTGACATCAAGGAGAAGC	TACCGCAAGATTCCATACCC
